# A Treatise on the Formation, Constituents, and Extraction of the Urinary Calculus; Being the Essay for Which the Jacksonian Prize, for the Year 1833, Was Awarded by the Royal College of Surgeons in London

**Published:** 1835-04-01

**Authors:** 


*A Treatise on the Formation, Constituents, and Extraction of the Urinary Calculus; being the Essay for which the Jacksonian Prize, for the Year* 1833, *was awarded by the Royal College of Surgeons in London.* By JOHN GREEN CROSSE, Surgeon to the Norfolk and Norwich Hospital.—London, 1835. 4to. pp. 231; Plates 29.

A WORK on Stone, by a surgeon of the Norwich Hospital, might at any time be expected to defy criticism; but, on the present occasion, when it has undergone the ordeal of the Council of the College of Surgeons, and been declared worthy of the prize, our task as reviewers merges into that of students, and we must be content to drink at the fountain, without venturing to give our opinion upon the taste or qualities of the waters. We trust our brethren will pardon us for descending from the altitude of universal knowledge, which *ex officio* belongs to us, nor consider us as traitors to our common dignity, in acknowledging that we can learn where it is our prerogative to dictate. Be that how it may, we are determined to risk the obloquy; and, regarding the text of this work as the record of very extensive experience, we shall venture on few observations, excepting in the way of comparison with the results of the metropolitan practice.

The labours of Drs. Yelloly and Prout have of late thrown so much light upon the formation and chemical composition of urinary deposits, that our author has not thought it expedient to dilate much upon these topics. With regard to the causes of calculous disorders, he agrees with all other writers, that they are generally consequent on dyspepsia; but he attributes more importance to sudden changes in the temperature of the atmosphere than, we believe, they are generally acknowledged to deserve. It is, in his opinion, to this cause that the county of Norfolk owes its unfortunate pre-eminence in the frequency of these affections; and, in support of this hypothesis, our author asserts that he has "repeatedly known persons, who were free from gravelly complaints whilst residing in the metropolis, affected by them, on spending a few weeks in the county referred to, and relieved, or entirely freed from them, on a change of residence, although in each situation they followed carefully the same diet." (P. 3.) There is no doubt much truth in this observation: every surgeon of experience must bear testimony to the sympathy between the skin and the urinary organs, especially in the diseased states of the latter; and also to the great relief afforded to patients suffering under this class of maladies, by preserving them from the effect of sudden atmospheric changes.

Of the local causes of stone, the principal are strictures of the urethra, enlarged prostate glands, diseased states of the mucous surface of the bladder, and hernial displacement of that viscus. In a note subjoined to the chapter, references are made to several cases where large calculous deposits have been consequent on cystocele; of which the most remarkable is a case published in the sixth volume of our respected predecessor, the London Medical and Physical Journal, where a stone, weighing twenty-three ounces, was found in the prolapsed bladder of a female, and presented an external tumour as large as a child's head.

Mr. Crosse has paid particular attention to the analysis of urinary concretions passed per urethram, and presents us with the following table of the relative frequency of different calculi.

| | |
|---|---|
| "Lithic acid or lithate of ammonia | 72 |
| Lithic acid and oxalate of lime | 9 |
| Oxalate of lime | 14 |
| Carbonate of lime | 1 |
| Triple phosphate | 2 |
| Fusible | 2 |
| Total | 100." |

The results pointed out in this table are satisfactory, first, as corroborative of the accuracy of Dr. Marcet's tables; and, secondly, as supporting the views of Dr. Prout as to the renal or vesical origin of different concretions. We need scarcely remind our readers, that this table is chiefly valuable as illustrative of the calculous disorders of Norfolk; Drs. Marcet and Henry having shown, by various tables, that their composition depends much upon locality. To prove this, we need only instance the mulberry calculus:

| | |
|---|---|
| In the Hunterian museum, they form | $\frac{1}{25}$ |
| In the Norwich collection | nearly $\frac{1}{4}$ |
| In the Guy's Hospital collection | more than $\frac{1}{4}$ |
| In the Manchester collection | $\frac{1}{4}$ |
| In the Bristol collection | $\frac{5}{12}$ |

Thus it may be seen that oxalate of lime may form either a very large or a very small portion of urinary calculi, according to the locality where the examination is instituted; and the same may be observed of all the other concretions, though not perhaps to the same extent. As, however, the tables from which these proportions are extracted were drawn up some years ago, it would be well if the surgeons of the various districts would follow the example of Mr. Crosse, and furnish us with the results of their more recent experience.

We are also indebted to our author for a more easy test of the oxalate of lime than that now in use: it is as follows.

"A particle of this concretion, being submitted to the flame of a spirit-lamp, urged by the common blow-pipe, a drop of dilute nitric acid is applied to the residue, and immediately globules of air are extricated, and can be seen rising through the fluid, with a magnifying glass, or even with the naked eye; these globules of air are carbonic acid gas, the heat applied having been just sufficient to decompose the oxalic acid, and out of its elements carbonic acid gas was formed, which united with the lime." (P. 8.)

The mechanical composition and growth of urinary calculi are becoming every day of greater interest, in proportion as the operation of lithotrity is more generally practised. Our author's remarks upon this part of his subject are excellent, and his plates still more so; but we must confess, though we hazard the charge of voracity, that we should have relished a greater abundance. Data as to the weight, friability, and form of the different calculi, are so especially required for the successful performance of that delicate operation, lithotrity, that we know of no other subject that would so well reward careful and assiduous research.

The friability of calculi is not always proportionate to their lightness or porosity: there are some composed of lithic acid, or lithate of ammonia and oxalate of lime, which, though extremely dense and heavy, are so fragile, that they may be broken either by some slight violence in sounding, or by shaking against one another in the bladder. Our author mentions an instance in which four calculi were fractured into twenty-two portions, under the action of the latter cause.

The rate of increase of different calculi is but little understood. Some surgeons have entertained the opinion that they are sometimes stationary; but, though instances of this kind may have occurred, they must be regarded as extremely rare. Those which increase most slowly are composed either of lithic acid or oxalate of lime. Our author's estimate is, that these may grow from one to two drachms yearly: he adds, however, that

"The actual increase, in all probability, will be greater, the larger the stone and more extensive the surface presented; but I have never found reason to believe that, in calculi of moderate size, above four drachms have been deposited in a year; the largest vesical calculi I have met with, weighing from eight to twelve ounces, have been fifteen or twenty years in forming. The following case demonstrates the rate of increase with some accuracy in a particular instance.

"A patient was lithotomized, and two lithic acid calculi, weighing seven drachms and a half, were removed, one of which broke into several portions under pressure of the forceps. The patient recovered from the operation, but soon had a recurrence of symptoms of stone, which he bore for above seven years, when he died aged seventy-six. The calculus found in his bladder weighed ℥ij. ʒij. ℈j., and presented on a section a clear exhibition of a portion of the calculus left in the bladder at the time he was lithotomized, and which formed the nucleus of the subsequent deposit. The calculus is composed of lithic acid, and about two ounces were deposited in the period of seven years and a half." (P. 12.)

We are of opinion that but little importance can be attached to this case brought forward by our author; not only because it is a single case, but because the details are not sufficiently minute. The composition of urine is constantly varying, as also is its degree of concentration and specific gravity; the process of crystallization (to which we believe the deposition of the stone to be similar,) cannot then go on at an equable rate. It is therefore evident that we must be furnished with a history of the state of the urine during the whole period, before we can form any guess as to the relative quickness of the increase of the calculus. It may have happened that, during the first portion of this time, (viz. immediately after the operation,) great attention was paid to the state of the urine, and it might have been kept in a nearly neutral state; whereas, afterwards the acidity was permitted to abound, and the deposition of calculous matter may then have been, out of proportion, rapid. It would be easy for us to point out other circumstances which might render the increase of calculi unequal; but, as they must present themselves to the minds of all our readers, we shall content ourselves with merely reiterating that, in order to form data by which we may judge of the size of the stone, from the duration of its residence in the bladder, we must be furnished with tables of the relative states of the urine and increase of the calculi. This our author has not attempted; but as, of itself, it is an ample subject of investigation, no blame can rest upon him for the omission. He has well performed his task, in accumulating so many facts as this splendid volume contains.

Calculi composed of the phosphates increased with greater rapidity than any others. Mr. Crosse relates a case, in which he operated, where a stone weighing four drachms and a half had been formed in little more than three months. We remember hearing a very celebrated surgeon, at a consultation, relate a case which occurred in his own practice, strongly illustrative of this fact; and, at the same time, conveying a lesson of caution to those who are fond of libelling their superiors. He had performed the operation of lithotomy, and extracted a stone composed of the phosphates. Almost immediately after the cicatrization of the wound, the symptoms returned, and the patient fell under the charge of another surgeon, who operated at the end of four months, and removed a very large calculus, of a similar character. It was soon noised abroad that the first-named surgeon had performed lithotomy, and left a stone in the bladder. He had, however, to endure the obloquy but a short time: the patient died at the end of a few weeks, and five phosphatic calculi were discovered in the bladder; leaving no doubt, in the minds of his medical attendants, that they had been formed since the operation.

Another circumstance, which it is important should be known, with reference to the increase of the size of a calculus, is, that two or more may be joined together by a deposit of fresh matter around them while lying in contact: this happened, as a note informs us, in the case of the largest vesical calculus ever known, it weighed forty-four ounces, and its history is given in the Philosophical Transactions for 1809, page 303.

Our author has collected some curious facts with regard to the existence of calculi in the kidneys and ureters. To establish sound pathological views is certainly the first step to improvement in practice; and, accordingly, our readers will need no excuse for our presenting them with the details of the only case on record in which oxalate of lime has been found in the tubuli uriniferi of the kidneys.

"An elderly man was treated for rheumatism and lumbago in a public institution, where he lost appetite, had a diminished secretion of urine, a parched brown tongue, and died in a few weeks. A calculus was found in the left ureter, seven inches from the kidney, completely preventing the passage of urine through that channel into the bladder, and inducing enlargement of the ureter above it, as well as of the pelvic cavity, which was filled with fœtid, muco-purulent fluid; the lining membrane of this cavity was thickened and morbidly vascular. The right kidney was of small size, but normal in exterior shape and in the condition of its pelvic and infundibular cavities. The parenchyma being cut through in different directions, the tubular part was found occupied by numerous white concretions, varying from the size of the smallest seeds to that of a large pin's head; these bodies were distributed over all parts of the substance of the kidney except the cortical portion; on more minute investigation, I found them to be pure oxalate of lime, crystallized, transparent, and situated in the tubuli uriniferi. I cut thin slices of the organ, dried them, and placed them afterwards in spirit of turpentine, which exhibited the small concretions of oxalate of lime distinctly; but no drawing will convey any adequate representation of them; one of the largest slices was prepared by drying, and before it was quite dry, I cut down to the calcareous mass, and thus opened one of the tubuli and exposed a beautifully crystallized salt of oxalate of lime, and in this state it is now preserved in my cabinet." (P. 16.)

Lithic acid has frequently been found in this situation; and indeed Mr. Wilson, in his lectures on diseases of the urinary and genital organs, affirms that he has found it in the cortical substance. This fact militates strongly against the hypothesis proposed by Dr. Prout as to the origin of renal calculi. This distinguished chemist supposes that, in the case of lithic acid calculi, the acid escapes from the tubuli uriniferi into the infundibulum in a semifluid condition, and that, after remaining in this state for a greater or less time, crystallization may perhaps take place either in small granules, so as to form red gravel, or in a larger mass, as the nucleus of a stone. In the instance of oxalate of lime, his theory is more completely disproved by Mr. Crosse's case; for he imagines that "a solution of oxalic acid, nearly in a saturated state, and in union with a little lime, is secreted by a portion of one of the kidneys, instead of the lithic acid in the former case; that this, enveloped in the usual animal matters, passes from the infundibulum into the pelvis of the kidney, and, there meeting with the lime naturally contained in the urine secreted by other parts of the kidney, instantly combines with it." (*Inquiry*, page 209, second Edition.) The fact of crystals of oxalate of lime having been found by our author in the tubuli uriniferi, of course, refers their origin to that structure. Dr. Prout's theory was highly ingenious, but the discoverer and elucidator of so many important facts may well afford to throw away a false hypothesis.

The symptoms of a calculus passing along the ureters are known to every practical surgeon, as well as their effect when they occasion obstruction of that tube, in dilating the pelvic cavity, and producing absorption of the structure of the kidney. It is not, however, as generally known that the passage of a calculus through the narrow part of one of the infundibula to reach the pelvis of the kidney may excite a very similar train of symptoms. Our author, nevertheless, has clearly established this fact by the relation of several cases, from which we select the following:

"A man, aged sixty-four, following the occupation of a dyer, complained for years of a pain in his loins reaching as high as the scapulæ, but was otherwise stout, strong, and healthy, and kept to his regular labour, until he was suddenly seized with more severe pain, attended by very obstinate vomiting; this latter symptom persisted for several weeks, at the expiration of which time, and after an entire suppression of the urinary secretion for five days, he died. On dissection I found the left kidney of great size, weighing when deprived of all adipose substance, above twelve ounces, and in the pelvic cavity, there was a large calculus, of the size represented in plate iv., fig. 1. The infundibula were deepened, but not greatly increased in size; and there were evidences of inflammation of the kidney and of the lining membrane of the pelvis, which I presume was the cause of the complete suppression of urine and of death; for this was the only kidney the patient had for performing the function of this emunctory, no other trace remaining of the right kidney than a lobulated bladder, filled with an opake dirty fluid. On inspection I found that the whole of the parenchymatous substance of the right kidney had been absorbed, the common lining of the pelvis and infundibula being brought into contact with the external capsular covering, in consequence of a small black calculus obstructing completely the ureter very near its commencement." (P. 19.)

Our author has devoted a chapter to the subject of Urinary Calculi situated in the Urethra, and on Calculous Concretions in the Prostate Gland. He opens it with an account of the symptoms produced by the lodgment of the stone in the prostatic portion of the urethra and the neck of the bladder, which are thus enumerated.

"A stone thus placed creates great pain, and is usually accompanied by constant stillicidium; it is easily felt with the sound, but this instrument meets with great obstruction when an attempt is made to introduce it into the bladder; indeed, if there have long been stillicidium, this viscus becomes so much contracted, that there is hardly a vesical cavity remaining to receive the end of the sound. The surgeon may recognise such a position of the stone not alone by the symptoms enumerated, but by the sound coming in contact with it before being passed deep enough to enter the bladder; and if the stone occupying the prostatic urethra be large, it can be felt by the finger introduced *per anum*." (P. 26.)

From this statement it would appear that these cases are of easy diagnosis: they are not, however, in some instances. We remember a case which occurred a few years ago, which misled some of the most eminent surgeons of the metropolis. The patient, who belonged to the middle ranks of life, applied to a surgeon of one of the London hospitals, celebrated for his success as a lithotomist. A sound was introduced, and came in contact with a stone at the neck of the bladder. The finger, introduced into the rectum, could readily feel the stone, but could not extend upwards to its limit: the quantity of urine retained in the bladder was extremely small. Under these circumstances the surgeon declined operating. The unfortunate sufferer applied to another surgeon of first-rate eminence, and expressed his extreme desire to undergo the operation, though the danger was fully explained to him. So convinced was the operator of the immense size of the stone, that he deemed it necessary to practise a new operation, and, in order to enlarge the aperture, to carry his incision backward, so as to lay open the rectum. On introducing the forceps, a stone was extracted, which proved to be of a moderate size, but of a shape somewhat resembling the os calcis; the anterior part being fixed in the urethra, while the larger portion projected towards the rectum. It is well that surgeons should be aware of the deception to which they are liable in such cases, lest, on the one hand, patients be left totally without a relief, or, on the other, lest the danger of the operation be needlessly increased. Mr. Crosse relates several cases of this kind; one in which the operation was unattempted, and another in which it was attended with difficulty, in consequence of the operator being ignorant of the real position and form of the stone.

The circumstances which render the diagnosis of these cases so difficult, is the constant stillicidium and great contraction of the bladder: this we presume to arise from the extreme sensitiveness of that triangular part named by the French *trigone vesical*, upon which the stone rests. For, when the calculus occupies only the prostatic or membranous portions of the urethra, without extending into the bladder, the symptoms are by no means so urgent; as the following case, extracted from Mr. Crosse's work, will demonstrate.

"About eight years ago, I attended in consultation upon a patient aged about fifty, on account of sudden retention of urine, and with the finger *in ano* I felt a calculus, of the size of a healthy prostate gland, situated in the membranous and prostatic part of the urethra; the patient would not submit to any operation for the removal of the stone, and is now living and in pretty good health, having suffered only from occasional retention of urine or from a frequent call to evacuate it. In other similar instances I have known so little suffered, that the removal of this stone seemed not to be demanded." (P. 29.)

When, however, these cases are complicated with stricture, they may sometimes prove fatal; but such is not their general termination: Nature will sometimes accomplish a cure by the formation of an abscess, which bursts externally, and affords room for their exit; and in other instances the surgeon, by the aid of Weiss's forceps, is enabled to extract them by the urethra, or by an incision in the perineum, to remove them from below. This latter method is more especially applicable where the calculus has made its way into the membranous portion of the urethra, and is there arrested. Cases of this kind are on record, and are referred to by our author, where the operation has been so easy, that patients have performed it successfully on themselves.

The origins of urethral and prostatic calculi are different: the former, though found in the prostatic portion, are generally of renal formation; the latter arise in the ducts of the prostate gland, and do not depend for their increase on the contact of urine, though they sometimes pass backwards into the bladder, and become the nuclei of vesical calculi. The following passage contains an important fact, with reference to calculous concretions in the veins, hitherto unnoticed by English pathologists, which must not be confounded with prostatic calculi, though of a similar composition.

"Concretions of another sort about the neck of the bladder ought to be noticed. In aged persons, particularly with hypertrophy of the prostate gland, a bladder diseased, and the veins about it and about the rectum varicose, concretions of phosphate of lime, varying in size from a pin's head to a kidney-bean, are often found in the veins; sometimes they present the appearance of a white pea, as in fig. 6, (b) of plate ii., and an inequality or projection is observable (c) answering to the surface by which the body adhered to the coats of the vein. These concretions have no connexion with the urinary or any other excretions, and should not be regarded as calculi; they are a morbid growth from the coats of the vein, to which at an early period they are invariably adherent, and a membrane covers them, upon the surface where not adherent, which I presume is the extended inner coat of the vein, the morbid growth originating in the outer coat. Fig. 6, (a) of plate ii, shews a portion of vein containing one of these concretions, and d, e, f, g, exhibit them of different shape and size; their chemical composition is chiefly phosphate and carbonate of lime, and they approach nearer to ossifications than to calculous concretions. I remember that Professor Meckel* has well represented them, but know of no English author from whom they have received the same attention." (P. 36.)

The chapter upon Calculi in the Urinary Bladder, and

"* Tabulæ Anatomico-Pathologicæ, auctore T. F. Meckel. Fasc. ii., tab. xiv., fig. 4, 5. Tiedemann, Otto, and Lobstein, have treated upon these concretions, and met with them in the veins of the uterus, vagina, and spermatic cord."

their pathological effects, contains little new general matter. For this, however, our author is not so much to blame as his predecessors, who, by their great attention to this part of the subject, have left but little to future investigators: we shall therefore confine our notice of this part of the subject to a single quotation.

"Stricture of the urethra is more often a cause than a consequence of stone in the bladder; but sometimes inflammation extends from this organ to the urethra, and severe permanent stricture is produced at the membranous part; at other times a stricture from inflammation is suddenly produced. A patient in advanced years suffered inflammation of the lining membrane of the bladder, apparently in consequence of numerous small concretions of lithic acid lodging in it, after descending from the kidney; the inflammation extended to the urethra; there was complete retention of urine, and difficulty in introducing an instrument. A surgeon, in rude attempts with the catheter, made a false passage anterior to the prostate, and the patient dying, I found the state of parts represented in plate xii., fig. 1." (P. 39.)

The plate displays a very narrow contraction of the passage along the membranous and prostatic portion of the urethra, with a lateral false passage.

Our author has devoted a larger space of his work to the method of sounding for stone than is usually deemed necessary: this detail will, however, be prized by the practical surgeon, who is aware of the extreme difficulty of discovering small calculi or fragments in the bladder, or of distinguishing these cases from tumours of different kinds, that are not unfrequently found there, and occasion very similar symptoms. In order to exemplify this a case is related, in which our author operated upon a boy, under the supposition that a stone was present, but proved that the symptoms depended upon numerous polypoid growths from the inner surface of the bladder. It is with great reluctance that we abstain from quoting this case, on account of the great length at which it is detailed: we can assure our readers that it is highly interesting, not only from the presence of mind evinced by the operator, but from the honourable frankness with which the error, and its fatal consequences, are narrated. We were once present at an operation, where, in addition to the stone which was extracted, a small polypus was discovered at the neck of the bladder. This was removed by the operator with a pair of scissors, and the patient recovered. Had it been overlooked, and the wound been suffered to heal, the symptoms of stone might have remained, and the sound coming in contact only with the soft tumour, no operation might have been again attempted for his relief. This complication should warn surgeons never to remove the patient from the operating table, without examining, as far as is practicable, the inner surface of the bladder.

We shall make no apology to our readers for presenting them with many of our author's directions for sounding.

After describing the shape of the sound, and directing that it should be of a moderate size, with a large well-polished handle, he thus proceeds:

"Children are never sounded in the erect posture; but when you have to deal with an adult patient, it will be found advantageous to make the first examination in that posture; in doing this, you should, with as little preparation and alarm to the patient as possible, introduce the sound lightly and gently, with a very delicate hand, endeavouring to steal as it were through the passage, by employing scarcely more than the weight of the instrument to propel it along and elevate its extremity into the bladder; if the operation be thus feelingly and judiciously managed, in nine instances out of ten, when there is a loose stone of any considerable size in the bladder, it falls down to the neck of this viscus, and is felt on the sound first entering. By alternately depressing between the thighs, and elevating the handle of the curved sound, you vary the extent to which it projects into the bladder, and will often in this movement feel a grating of the calculus against the instrument; the impression thus obtained is usually obscure and seldom to be relied upon. With the handle depressed more or less, and held centrally, answering to the median line of the body, you may jerk it upwards and backwards towards the rectum, when it will strike a stone lying in that direction, producing a sensible resistance and often also an audible sound, satisfactory evidences, when conjoined, of a calculus being present. You may give the same movement to the sound, with the handle inclined more or less to one groin, and thus explore each lateral, as well as the posterior part of the bladder. Should a stone not be felt under these movements of the sound, you may suspect it to be on the pubic or concave side of the instrument, when it will be felt by your drawing the instrument downwards and forwards, which movement should be performed first with the handle answering to the median line and more or less depressed between the thighs, and afterwards, with it inclined obliquely to either side, which will explore the lateral and anterior part of the cavity; if in any of these trials, the stone be felt, touching the concave side of the sound, you know it to be situated towards the os pubis; you may also, with the curved sound projecting considerably into the bladder, turn the handle to some extent upon its own axis, making its extremity describe a part of a circle, and sweeping the upper and lateral parts of the bladder. By a practised hand, the sound is in a short time made to perform these different movements, and the object is, by a regular succession of them, to carry the sound to every part of the vesical cavity.

"Where a careful sounding is required, the patient should be placed horizontally on his back; indeed the surgeon should always bear in mind how advantageous it is to vary the position of the patient, and how much may be gained by so doing. If, when the patient is standing, the stone be felt on the pubic side of the instrument, and when dorsally recumbent, you find the stone behind it, towards the rectum, you know it to be moveable and loose. The sound being in the bladder, the shoulders of the patient may be raised into the half-sitting posture, or they may be depressed greatly so as to have the pelvis on the top of an inclined plane and make the axis of the spine answer to an angle of forty-five degrees, this latter being the method to remove the stone from the neck of the bladder and carry it to the fundus; he may likewise be placed on either side, or upon his hands and knees with the face downwards; all these changes of position should be made after the sound is introduced; the last is particularly applicable to cases of enlarged prostate gland, behind which there is a cavity not accessible to the long curved sound by any movement that can be given to it; and you can only remove this defect by changing the situation of the stone in the bladder, which is accomplished by altering the position of the patient.

"Where the sound touches the stone in different directions, and is found to pass over a large surface of it, you may conclude it is of large dimensions; but when, under the same position of the body, you do not feel it repeatedly, on passing the sound to the same part of the vesical cavity, it is likely to be small." (P. 51.)

Our author insists on the propriety of examining by the rectum, and gives copious directions for the conduct of this part of the exploration. We think, however, that he attaches too much importance to it, as it is applicable only to young patients, in whom the stone may be easily detected and examined by the sound. He differs also from Sir Everard Home and Sir Benjamin Brodie, as to the utility of the gum elastic catheter, in affording evidence of the presence of a stone. Now, it is well known that the former of these surgeons preferred the gum catheter to any other instrument, and yet was eminently successful in detecting a calculus in the bladder; and we should therefore be inclined to doubt whether Mr. Crosse's opinion does not mainly depend upon his having but rarely employed it.

The chapter upon Extracting small Vesical Calculi by the Urethra contains much valuable information concerning the method of employing Sir Astley Cooper's instrument for this purpose. Our author prefaces his observations with a detail of the symptoms attendant on the presence of small calculi in the bladder; and, as every year the diagnosis of these, in their earliest stages, becomes of more importance, we shall quote the passage containing their description.

"Where symptoms of stone have not existed above two or three months, or have been absent for a time and suddenly returned in a severe degree, producing itching at the end of the penis, frequent painful micturition, and occasional retention of urine, we may suspect that a small calculus is present in the bladder. With a small stone, the patient is often free from all inconvenience for a day or two, and even a week or two, and then is suddenly seized with retention of urine and most distressing pain, from the calculus entering the commencement of the urethra. In the interval between these sudden and acute attacks, the patient experiences only a slight itching at the end of the penis, irritation about the neck of the bladder, and a more frequent and sudden call to pass urine than is healthy. On sounding at this early period, you will occasionally find such an audible click, or noise produced, when you strike the stone, as can be heard at a distance of several yards: and the evidence thus obtained, more audible than tangible, arising from a clear and sharp sound, I have experienced only when the stone is small." (P. 57.)

"Perhaps the best indication we can get, of a vesical calculus being of small size, is to have traced it, not long before, passing through the ureter from one of the kidneys; but this source of information is rarely afforded, and the surgeon must trust to, and form his judgment upon, the reported duration of the symptoms, the preceding and present degree of their intensity, and the evidence derived from sounding the bladder. If the symptoms have steadily persisted, in a severe degree, for six or eight months, if the concussion of walking or riding produce pain in the glans penis or occasionally render the urine bloody, if there be a burning heat at the end of the penis, continuing some time after each evacuation of the bladder, the stone may be regarded as of too large a size to be brought through the urethra, and the urethro-vesical forceps ought to be very guardedly, if at all, employed; and when sounding comes in support of the opinion that there is a calculus of considerable size present, as pointed out by the dull noise, firm resistance, and extent of surface touched, the urethro-vesical forceps should on no account be introduced." (P. 59.)

Excellent as is this description, we would advise our readers not to be content with it alone, but to peruse the account of the early symptoms of stone given by Baron Heurteloup, in his Principles of Lithotrity. We should ourselves feel inclined to quote them, were it not that we have still much matter before us, which requires to be considered at length.

Mr. Crosse's directions for the use of Sir Astley Cooper's instrument are excellent, and the case which he relates of the removal of nine calculi by the urethra embodies many practical hints as to the method of operating. We would direct the attention of surgeons to these, as the operation is not so simple, or so free from danger, as may be supposed. Many instances have come to our knowledge where operators have been foiled in their attempts; and even one which terminated fatally, from the violence employed in endeavouring to withdraw the stone, which lay far down between the blades of the instruments.

There is a modification of this operation mentioned by Mr. Crosse, on which we would venture a few observations, viz. drawing the stone into the urethra, cutting down upon it, and removing it through the wound. This is a good operation, within proper limits, and should be attempted wherever the stone is sufficiently small to enter the membranous portion of the urethra. It is then a safe and easy operation, and unattended with any permanent inconvenience. It may, however, sometimes happen that the stone will pass as far as the spongy portion: is it then to be removed by incision? Our author says yes, and has performed the operation. We, however, are inclined to differ from him, as an incision "just anterior to the scrotum" is frequently attended with troublesome hemorrhage, and followed by a fistulous opening, very difficult to heal. We should rather recommend an attempt to break the stone in that situation; or, that failing, to push it back into the bladder, and, introducing the screw lithotrite of Weiss, to draw the stone into the membranous portion, there crush it, and allow the fragments to be washed away by the stream of water.

We now arrive at the portion of the work devoted to the operation of Lithotomy, a word for which our author would substitute Litho-cystotomy; but, as we think his nomenclature is not likely to be generally adopted, we shall not enter into any disquisition upon the propriety of adhering to old names, which are generally understood. The method of performing the lateral operation with the straight staff having been strongly recommended to the profession, not only by Mr. Key's work, but by his skill and success, requires the consideration of every surgeon, before he decides upon any plan for his own practice; and, as Mr. Crosse coincides with that distinguished operator, it will perhaps not be out of place if we make some observations upon the comparative merits of the straight and the curved staff. The advantages of the straight staff are thus stated by our author:

"The straight staff, in passing through the membranous part of the urethra, lifts it up from the rectum, pressing against the pubic or superior surface of the passage, thus affording great protection against wounding the rectum; the reverse happens with the curved staff, its convexity pressing towards the rectum, and rendering it not easy always to avoid wounding it. The greatest gain from the straight staff is in the facility given to the third stage of cutting into the bladder, by the instrument answering to the median line at the same time that its groove is presented in the most favorable position, and by your having to cut in a straight direction; so that, getting down to the staff, you find this third stage converted into one plain continued incision, effected by carrying on the knife in the groove, as you would carry it along a common director, till satisfied that you have gone as deep as required, passing the prostate gland and just entering the bladder; you then enlarge the incision in withdrawing the scalpel." (P. 74.)

The removal of the urethra from the rectum, and the straight incision into the bladder, are the advantages proposed by the advocates of the straight staff; and to a beginner it may be doubtful whether such advantages are to be slighted. But, it may be asked, how often do experienced operators wound the rectum, or fail in cutting into the bladder. Did either of these accidents occur to the late Mr. Martineau, whose practice our author had such excellent opportunities of witnessing? or are they common among the numerous dexterous surgeons of our London or county hospitals? If not, the whole credit of the new operation must rest upon this single fact, that, to those who do not know how to operate, it presents fewer difficulties than the old plan in the third stage of the operation. On the other hand, the objections to it are numerous: First, the difficulty of introducing a straight instrument in many cases, especially those with a large prostate gland; and the mischief sometimes done to the parts by the violent dragging of such an instrument, when forced into the bladder. Secondly, that the surgeon must be prepared to perform the old operation, as, in the before-mentioned cases, the new is inadmissible; and he is therefore obliged to acquire the power of performing both, instead of one. Thirdly, the new operation substitutes mechanical facility for anatomical knowledge; since the straight staff, by removing the various structures from their natural position, places the scientific operator on the same footing as the more ignorant one, and does away with the necessity of studying the relative anatomy of the parts. Lastly, the straight staff cannot generally be brought in contact with the stone, nor retained there during the operation; so that the process of removing the stone from the bladder is rendered much more difficult; and, as far as our own experience goes, this is the stage in which the young operator is most likely to be foiled, when he has no guide to the position of the stone. Such are briefly the objections to the straight staff: nor is it sufficient answer, that, in the hands of a Key or a Crosse, it is eminently successful, unless, at the same time, it be conceded that the cutting gorget (the use of which is now almost abandoned,) is to be recommended on account of the skill of its inventor, Sir Cæsar Hawkins; or that all modern improvements are to be abandoned in favour of the apparatus of Cheselden, the most fortunate operator, perhaps, that ever existed.

But enough has been said upon this subject; let us now follow our author to some of the steps of the operation; and first we shall present our readers with his directions for holding the staff. He says (and we think with the greatest truth,) that the operator is dependent for his success on the staff holder. This cannot be impressed too strongly upon the minds of those who are called upon to assist at operations for the stone; and it is to be regretted that surgeons are too often content with studying how to operate themselves, to the total neglect of that equally important, though less brilliant, part of their professional duties, which consists in assisting another. Our author appears to have felt the want of such assistance, and he therefore is minute in his directions.

"The staff holder is the operator's main assistant, who should previously understand his views, and sympathise with him in every step of the operation. I have felt myself so dependant on such an assistant, that, preferring my fate to be in my own hands, I have sometimes wished to imitate Pouteau, by holding the staff for myself; but I have never undertaken to do so. This instrument must be held not forcibly, but lightly and as if suspended in air, since pressing it towards the sacrum to steady it, or pulling it towards the pubes, will equally tend to embarrass the operator and create mischief; it should be kept in the same relative position, in regard to the patient, as that in which it was received from the hands of the operator. Besides the danger of pressing the staff towards either rectum or pubes, its holder, in endeavouring to make its convexity prominent in the perinæum, may cause its extremity to desert the bladder, so that it reach no deeper than the prostatic part of the urethra. By the unsteadiness of the patient, the staff may be moved irregularly from side to side, or backwards and forwards in the urethra; to prevent all this, the patient's pelvis must be kept steady, and if it move, there must be a corresponding movement of the staff, that it may retain the same relative position in regard to the patient's body." (P. 72.)

After describing the mode of making the external incision, and of cutting down upon the staff, he proceeds to show how the incision should be made into the bladder. Now, our readers are aware that surgeons of the present day differ much as to the size of this incision; some thinking, with Sir Benjamin Brodie, that it should be small, and that the prostate should be split by the blunt gorget; others, with Mr. Fletcher, of Gloucester, that it should be large, as any sized incision is safer than violence. Our author seems to hold a middle course: he recommends that it should be free, but speaks also of its being requisite to dilate the wound generally, in order to withdraw the stone. It is probable that our author is nearer to the truth than either of the others, as it appears, from a note, that the last thirty-eight patients operated upon at the Norwich hospital in this method have all recovered, and that it had the sanction of the late Mr. Martineau, who in this operation has had few rivals. Our author thus sums up his directions.

"In recapitulating what belongs to this part of the operation, which I have attempted, however imperfectly, to describe, the best rules I can lay down are, to make the deep incision, through the prostate gland and neck of the bladder, of moderate extent, so that the forceps may readily enter, to dilate a little with the finger and forceps, before grasping the stone, to act slowly in the extraction, that the wound may still further dilate, and to enlarge the wound without hesitation, and to the requisite extent, if you find great resistance to the passage of the foreign body to be removed; thus, you will have the best chance of accomplishing a safe, though you may fail to have a rapid and brilliant operation. The quickest operations of litho-cystotomy, in my experience, have not usually proved the most successful. The dissecting-room rule, which all young operators are ready to put in practice, of cutting freely so as to effect a rapid operation and meet with little or no resistance to the extraction of the stone, is not sanctioned by the experienced and practical teacher. Gentleness and precision ought rather to be studied than great expedition. Le Cat cut about half a dozen patients in twice as many minutes, and, it is said, lost nearly all of them! At any rate, whether the operator cut much or little, let him not use the forceps boisterously, but temperately and with gentleness, for violence, if it do not produce immediate laceration of soft parts, will be sure to bring on subsequent inflammation, tumefaction, and sloughing.

"In children the parts at the neck of the bladder dilate so readily, and bear to be dilated so well, that if the operator can get the forceps into the bladder, and the stone be of moderate size, he may remove it safely, although the incision have failed to reach so deep as it ought; but in adults, and particularly in the aged, the soft parts will not so yield, and force applied will lacerate, creating fatal injury." (P. 77.)

The most frequent cause of death after lithotomy is undoubtedly diffuse inflammation of the deep cellular tissue, from infiltration of urine. This is, however, a discovery of modern times, and the treatment which it demands is not generally understood. Bleeding is, we believe, fatal in such cases, unless the disease has gone so far as to excite peritoneal inflammation, which must be considered as the second stage. Even then, however, it should only be local; though perhaps it matters but little what is done, as the patient almost invariably dies. Generous diet, and a free outlet for the sloughing cellular membrane, are the only means worthy of trust. For some excellent observations on the after-treatment of the wound, we must refer our readers to the work itself.

The last chapter of the work is upon "Hemorrhage after Litho-cystotomy," and the principal part of its contents apply to arterial hemorrhage. With regard to the treatment of this unfortunate accident, our author recommends that, if a vessel of any size be wounded in the first two stages of the operation, it should be secured by a ligature; but, if in the third, the remedy must be suited to the particular vessel. If it be the artery of the bulb or the internal pudic, these may also be reached with the tenaculum, and tied like the others, with this single precaution, that, in the case of the latter, both ends of the vessel must be secured, in order to stop the bleeding effectually. But, should the vessel be situated deep down, on the inner surface of the levator ani, if pressure upon the internal pudic artery be insufficient to command it, our author recommends that the wound should be plugged with lint around a hollow canula. He confesses, however, that this method is dangerous, as the blood sometimes flows into the bladder, and occasions serious symptoms. Let it be permitted to us to recommend a method, which we have seen employed by Sir Benjamin Brodie, with the happiest effects, as more easy of application, and unattended with danger.

A caoutchouc female catheter should be introduced through the wound into the bladder, in order to give free vent to the urine, while an assistant, with his finger in the rectum, makes pressure on the different parts wounded, until he discovers the point at which the bleeding takes place: of course, the pressure must be continued until all hemorrhage has ceased. We have never seen this plan fail, and it has none of the disadvantages which accompany violence to the wounded surface.

Though we have now given rather a copious abstract of the work, we should do but scanty justice to its merits, were we to conclude without mentioning the plates. Their great number, their large size, and the accompanying explanations, contribute to make them, perhaps, the best representations extant of diseases of the bladder. He who shall carefully study them will reap an ample reward for his labour. Besides these plates, there are two appendices, which did not form part of the prize essay: the former consists of cases which have occurred under our author's notice, and which are of the highest interest; the latter contains tables of the result of the operation of lithotomy at the Norfolk and Norwich hospitals. Mr. Crosse has presented us with a list of one hundred fatal cases of lithotomy, showing the age of the patient, the weight of the calculus, and the interval between the operation and death. If he would add a sketch of the cause of death, it would enable us to judge accurately of the period at which a patient escapes certain dangers, and becomes liable to others.

The work concludes with a catalogue of the treatises upon Gravel, Stone, and Lithotomy, published in different ages and countries, and of essays or notices referring to those subjects in many periodical works. Of this part we can say nothing further than that it is worthy of the author, who must have perused no inconsiderable portion of the books to which he refers, in order to enrich his text with so many references to parallel cases. In short, we may sum up by observing, that experience and study have done their utmost for this work, and that its form alone will prevent its circulation from being equal to its merits.

